# Efficacy of tocilizumab in anti‐ *N* ‐methyl‐ d‐asparate receptor encephalitis with Graves' hyperthyroidism and positive anti‐glial fibrillary acidic protein antibodies

**DOI:** 10.1111/cns.13949

**Published:** 2022-08-26

**Authors:** Yin‐Xi Zhang, Meng‐Ting Cai, Er‐Chuang Li, Yong‐Feng Xu

**Affiliations:** ^1^ Department of Neurology, Second Affiliated Hospital, School of Medicine Zhejiang University Hangzhou China

**Keywords:** anti‐glial fibrillary acidic protein antibody, anti‐*N*‐methyl‐d‐asparate receptor encephalitis, Graves' hyperthyroidism, tocilizumab

Dear Editors,

Anti‐*N*‐methyl‐d‐asparate receptor (NMDAR) encephalitis, mainly characterized by neuropsychiatric symptoms, is the most common subtype of autoimmune encephalitis affecting predominantly females of a childbearing age.^1^ Patients with anti‐NMDAR encephalitis have been identified to coexist with multiple autoantibodies and/or combine with some other autoimmune diseases in clinical practice, which are great challenges to diagnosis and treatment. Here, we report the first case of anti‐NMDAR encephalitis positive for anti‐glial fibrillary acidic protein (GFAP) antibodies with concomitant Graves' hyperthyroidism. First‐ and second‐line immunotherapy failed to bring satisfactory effect, but further tocilizumab treatment led to significant improvement.

A previously healthy 32‐year‐old female developed recurrent fever with fatigue and headache for 3 months. Abnormalities in thyroid parameters were detected in the local hospital (Table 1). Thyroid ultrasound showed bilateral thyroid enlargement with diffuse changes in echogenicity. Graves' hyperthyroidism was diagnosed and methimazole was administrated. One month after, psychosis including anxiety, agitation and bizarre behavior emerged. Hyperthyroidism‐induced psychiatric disorder was considered. Symptomatic treatment was performed with an unsatisfactory outcome. She had an intermittent low fever, reduction of speech, and seizures, which almost occurred daily.

**TABLE 1:** Serum thyroid parameters (thyroid function/antibodies) in different stages

Parameter	Onset in the local hospital	Admission to our hospital	At 1‐year follow‐up	Reference range
FT3 (pmol/L)	12.96 ↑	5.6	3.91	2.43–6.01
FT4 (pmol/L)	40.38 ↑	22.25 ↑	12.39	9.01–19.05
TSH (mIU/L)	<0.004 ↓	<0.01 ↓	1.56	0.35–4.94
TPOAb (IU/ml)	95.34 ↑	22.05 ↑	4.22	<5.61
TGAb (IU/ml)	18.11 ↑	2.76	0.54	<4.11
TRAb (IU/L)	12.62 ↑	NA	1.57	<1.75

Abbreviations: FT3, free triiodothyronine; FT4, free tetraiodothyronine (thyroxin); NA, not available; TGAb, anti‐thyroglobulin antibody; TPOAb, anti‐thyroid peroxidase antibody; TRAb, anti‐thyrotropin receptor antibody; TSH, thyroid‐stimulating hormone.

The patient was then transferred to our hospital. On admission, she was tachycardic, had a low fever and diffusely enlarged thyroid. Neurological examination revealed confusion, slurred speech, and the others could not be evaluated or negative. The modified Rankin Scale (mRS) score was 4. Encephalopathy associated with autoimmune thyroid disease (EAATD) was suspected.

Routine and specialized laboratory workups including rheumatology and immunological panel, tumor markers and infectious screening were unrevealing, except for thyroid parameters (Table 1). Contrast‐enhanced magnetic resonance imaging (MRI) of the brain, computed tomography of chest and abdomen, ultrasound of the pelvis showed no abnormalities. Cerebrospinal fuid (CSF) analysis was unremarkable. Further investigation for neural antibodies demonstrated positive antibodies against NMDAR and GFAP in CSF, both with an elevated titer of 1:32 using cell‐based assay. A modified diagnosis of anti‐NMDAR encephalitis positive for anti‐GFAP antibodies with concomitant Graves' hyperthyroidism was made.

The patient was treated with intravenous methylprednisolone (daily 500 mg for 5 days, followed by a tapering scheme) combined with intravenous immunoglobulin 0.4 g/kg per day for 5 days. Adjuvant symptomatic treatments such as anti‐seizure medications were also given. Rituximab was subsequently initiated (two infusions of 1000 mg given a fortnight apart). The patient's symptoms alleviated with the mRS score of 3. In the following 3 months, the patient failed to achieve further improvement and the mRS score remained 3. Re‐examination showed positive anti‐NMDAR antibodies (titer 1:3.2) but negative anti‐GFAP antibodies in CSF. Rituximab was replaced with tocilizumab at a dose of 8 mg/kg monthly. At 1‐year follow‐up, the patient completed five courses of tocilizumab treatment. Re‐test revealed a continuously decreasing titer (1:1) of anti‐NMDAR antibodies with negative anti‐GFAP antibodies in CSF, and normal thyroid indicators in serum (Table 1). She only had mild slurred speech and went back to normal work. The mRS score turned to 1 (Figure 1).

**FIGURE 1:**
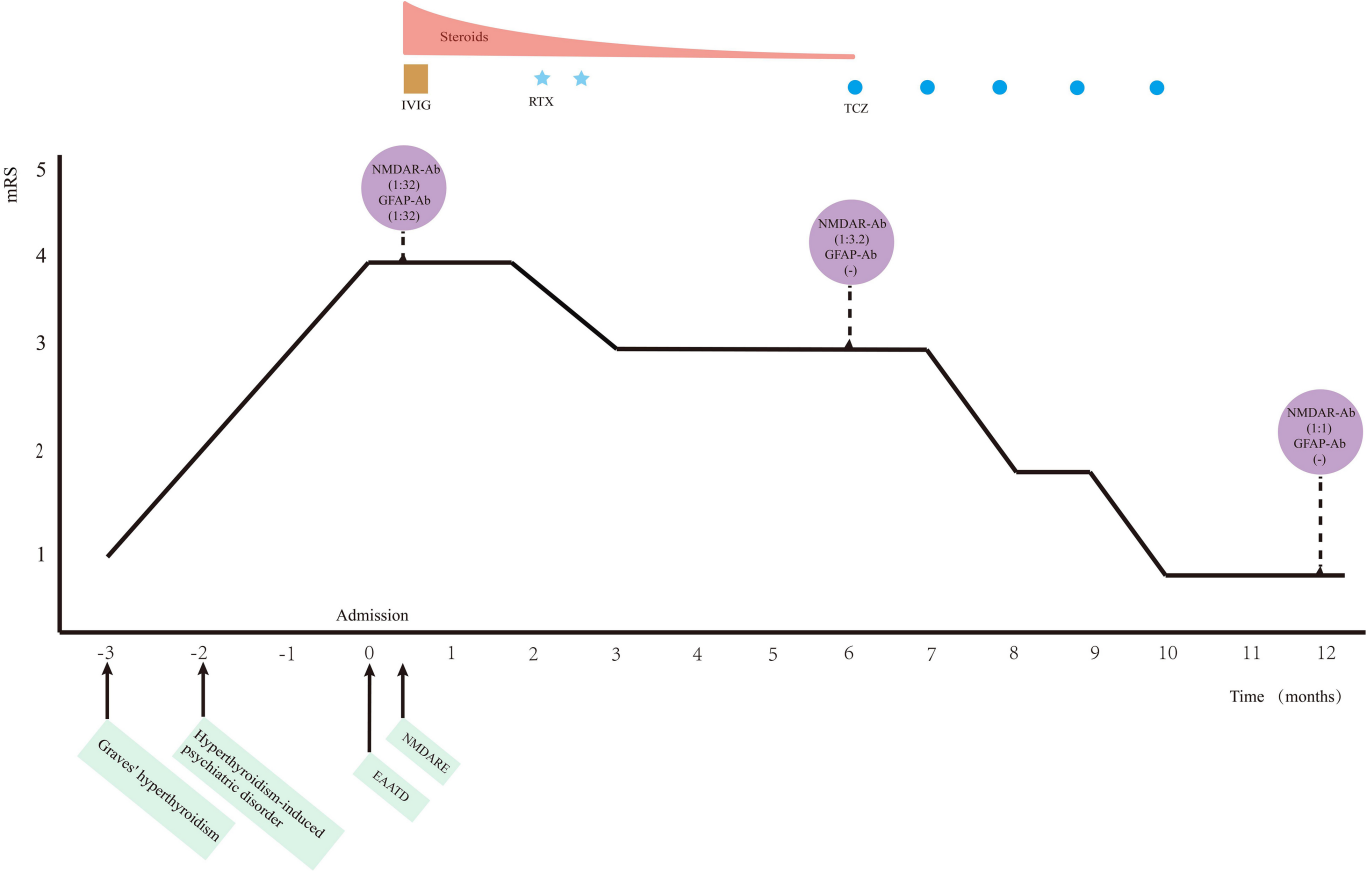
Timeline of the diagnosis and immunotherapy in the case. Timeline depicting patient neural antibody detection, mRS score, diagnosis and immunotherapy. For further details, refer to the main text. Ab, antibody; EAATD, encephalopathy associated with autoimmune thyroid disease; GFAP, glial fibrillary acidic protein; IVIG, intravenous immunoglobulin; mRS, modified Rankin Scale; NMDAR, *N*‐methyl‐d‐asparate receptor; NMDARE, anti‐*N*‐methyl‐d‐asparate receptor encephalitis; RTX, rituximab; TCZ, tocilizumab.

This young female patient had a complicated diagnostic and therapeutic process. Although hyperthyroidism can be accompanied by psychiatric manifestations, it is common in patients with pre‐existing psychiatric disorders and rarely induces acute psychosis.^2^ The case reported here developed psychiatric abnormalities during the anti‐thyroid therapy with improved thyroid parameters. Moreover, her symptoms progressed with new‐onset presentation, indicating further assessment to explore potential causes.

EAATD, also known as steroid responsive encephalopathy associated with autoimmune thyroiditis or Hashimoto's encephalopathy,^3^ is classified as probable autoimmune encephalitis.^4^ One of the diagnostic criteria for EAATD is the absence of well‐characterized neuronal antibodies in serum and CSF. In the current case, positive anti‐NMDAR antibodies were detected in keeping with definite anti‐NMDAR encephalitis.^4^ EAATD was then excluded.

It is reported that approximately 70% of patients with anti‐NMDAR encephalitis have prodromal symptoms, such as headache and fever. Psychiatric symptoms are often the initial manifestations in adult patients, and seizures are frequently observed in the acute phase.^1^, ^5^ Moreover, various symptoms, including reduction of speech, memory deficit, dyskinesia, consciousness impairment, and autonomic dysfunction, may also appear in the course of disease progression. Consistently, the case reported here had a series of clinical symptoms typical of anti‐NMDAR encephalitis in succession. Unlike other common antibody‐mediated encephalitis, more than half of patients with anti‐NMDAR encephalitis have normal neuroimaging with routine MRI sequences,^1^ as also seen in our case. A recent network‐based study demonstrated that anti‐NMDAR encephalitis caused abnormalities in brain morphological and structural networks, indicating multimodal MRI evaluation might contribute to objective diagnosis.^6^

Notably, the patient had two autoimmune diseases simultaneously. Previous studies reported that anti‐NMDAR encephalitis could be discovered with other concurrent autoimmune diseases or other autoimmune alterations.^7^, ^8^ Nevertheless, anti‐NMDAR encephalitis combined with Graves' hyperthyroidism is extremely rare.^2^ Our case indicated that there might be a possible link between these two diseases, suggesting a shared mechanism of autoimmune pathophysiology. Recognition of these associations is of great significance to avoid misdiagnoses and refine the treatment.

Interestingly, anti‐GFAP antibodies, specific biomarkers of autoimmune GFAP astrocytopathy,^9^ were also detected in the CSF of this patient. Our case showed no distinctive linear radial perivascular gadolinium enhancement or any other abnormality on brain MRI. Furthermore, the anti‐GFAP antibodies in CSF turned negative instantly after treatment. Thus, bystander (secondary) phenomenon of anti‐GFAP antibody was assumed.

For the treatment, we tried tocilizumab, a humanized monoclonal antibody against interleukin‐6 receptor, when second‐line rituximab treatment failed to achieve continuous remission. Our patient responded well to tocilizumab and had a decline in mRS score from 3 to 1 after 5 courses of therapy. The long‐term prognosis requires further follow‐up observation. More cases are needed in the future to better confirm the efficacy of tocilizumab in the treatment of anti‐NMDAR encephalitis with Graves' hyperthyroidism.

Our case broadens the clinical spectrum of this treatable disease and indicates that the concomitant multiple autoantibodies and autoimmune diseases might share pathophysiological mechanisms. In anti‐NMDAR encephalitis concurrently with multiple autoantibodies or combined with other autoimmune diseases and resistant or intolerant to conventional immunotherapy, tocilizumab might be an ideal treatment option.

## CONFLICT OF INTEREST

The authors declare that they have no conflict of interest.

## INFORMED CONSENT

Informed consent was obtained from the patient for publication of this paper.

## Data Availability

The data that support the findings of this study are available from the corresponding author upon reasonable request.
